# Sub-Sets of Cancer Stem Cells Differ Intrinsically in Their Patterns of Oxygen Metabolism

**DOI:** 10.1371/journal.pone.0062493

**Published:** 2013-04-30

**Authors:** Luke Gammon, Adrian Biddle, Hannah K. Heywood, Anne C. Johannessen, Ian C. Mackenzie

**Affiliations:** 1 Blizard Institute, Barts and The London School of Medicine and Dentistry, Queen Mary University of London, London, United Kingdom; 2 The Gade Institute, University of Bergen, Haukeland University Hospital, Bergen, Norway; 3 School of Engineering and Materials Science, Queen Mary University of London, London, United Kingdom; University of Cincinnati, United States of America

## Abstract

The glycolytic response of hypoxic cells is primarily mediated by the hypoxia inducible factor alpha (HIF-1α) but even in the presence of abundant oxygen tumours typically show high rates of glycolysis. Higher levels of HIF-1α in tumours are associated with a poorer prognosis and up-regulation of markers of epithelial mesenchymal transition (EMT) due to HIF-1α actions. We have recently shown that EMT occurs within the CD44^high^ cancer stem cell (CSC) fraction and that epithelial and EMT CSCs are distinguished by high and low ESA expression, respectively. We here show that hypoxia induces a marked shift of the CSC fraction towards EMT leading to altered cell morphology, an increased proportion of CD44^high^/ESA^low^ cells, patterns of gene expression typical of EMT, and enhanced sphere-forming ability. The size of EMT fractions returned to control levels in normoxia indicating a reversible process. Surprisingly, however, even under normoxic conditions a fraction of EMT CSCs was present and maintained high levels of HIF-1α, apparently due to actions of cytokines such as TNFα. Functionally, this EMT CSC fraction showed decreased mitochondrial mass and membrane potential, consumed far less oxygen per cell, and produced markedly reduced levels of reactive oxygen species (ROS). These differences in the patterns of oxygen metabolism of sub-fractions of tumour cells provide an explanation for the general therapeutic resistance of CSCs and for the even greater resistance of EMT CSCs. They also identify potential mechanisms for manipulation of CSCs.

## Introduction

Tumours are highly glycolytic even in the presence of abundant oxygen, the so-called “Warburg effect” [1], [2]. Hypoxia inducible factor 1 alpha (HIF-1α) is the major factor regulating cellular hypoxic responses [3]. At high oxygen levels, HIF-1α is ubiquitinated and targeted for degradation whilst at lower oxygen levels degradation is inhibited and HIF-1α translocates to the nucleus where it dimerises with hypoxia inducible factor 1 beta (HIF-1β) and binds to the hypoxia response elements (HREs) of target genes that aid cellular adaptation to hypoxia [4]. Overexpression of HIF-1α occurs in a wide range of primary and metastatic cancers [5], and is responsible for a range of tumour-related properties including a reduction in reactive oxygen species [6], increased radio-resistance [7]–[9], and protection of cells from drug induced apoptosis [10] and senescence [11].

Tumour invasion and metastasis have become increasingly associated with cancer stem cells (CSCs), a sub-set of cancer cells that is capable of self-renewal, has tumour-initiating ability, and is resistant to therapy [12], [13]. Both local tumour invasion and metastasis to distant sites require migratory abilities acquired through epithelial to mesenchymal transition (EMT) of CSCs [14] during which epithelial characteristics are lost and epithelial proteins such as E-cadherin are down-regulated and of mesenchymal proteins such as Vimentin and Twist up-regulated [15]. Induction of EMT in breast cell lines results in cells acquiring the marker phenotype typical of breast CSCs, greater motility, and resistance to therapeutic agents [16], [17].

In HNSCC and several other carcinomas, sub-populations of CSCs have high expression of CD44 [18]–[21]. We have recently shown that in cell lines derived from oral and skin carcinomas, EMT occurs within the CD44^high^ CSC fraction resulting in two CSC phenotypes, one that is epithelial and shows high expression of epithelial specific antigen (ESA), and another that has EMT characteristics and low expression of ESA [22]. CSCs can switch between the epithelial and the EMT phenotypes and both fractions initiate tumours after *in vivo* murine transplantation [22]. As several studies have now directly linked hypoxia and high HIF-1α to EMT [23]–[26], we wished to know whether innate metabolic differences related to oxygen utilization exist between the epithelial and EMT CSC phenotypes. We show that low oxygen levels reversibly increase the size of EMT fractions within HNSCC cell lines and that, compared with epithelial CSCs (Epi CSC), EMT CSCs have higher levels of the hypoxic response protein HIF-1α, even under normoxic conditions. There are also major differences in metabolism of this subpopulation with the higher levels of HIF-1α expression in EMT CSCs correlating with up-regulation of glycolytic genes, a marked reduction in oxygen consumption, decreased mitochondrial mass and membrane potential, and reduced production of reactive oxygen species (ROS).

## Materials and Methods

### Cell Culture and Hypoxic Induction

HNSCC cell lines were grown as previously described [19]. With the exception of H357 [27], all cell lines (Ca1, LuC4, CaLH2, CaLH3) and normal oral keratinocytes (NOK2 & NOK3) were derived in our laboratory. Tissue was collected with written informed consent following a protocol (Oral Cancer, 04/Q0601/53) approved by the NE London & The City Ethics Committee. Hypoxic induction involved culturing cells in an InVivo1000 hypoxic chamber (Ruskinn Life Sciences, Wales, UK) at 0.2% or 2% O_2_ with 5% CO_2_. HIF-1α stabilization used 1 mM Dimethyloxalylglycine (DMOG) (Sigma) and inhibition 3-(5′-hydroxymethyl-2′-furyl)-1-benzylindazole (YC-1) (Sigma) at 10 µM or 50 µM. For sphere formation assay, plates were coated with PolyHEMA (Sigma) (12 mg/ml in 95% ethanol) to inhibit attachment of cells plated at 1000 cells/well in FAD medium with the addition of 1% methlycellose (Sigma). TNFα treated cells were cultured for 6 or 24 hours with 10 ng/ml recombinant TNFα (R&D systems Cat# 210-TA-010).

### Western Blotting

Western blotting was preformed as previously described [28]. Protein quantification used a Bradford assay and antibodies and dilutions were as follows; HIF-1α – Mouse 1∶1000 Cat# 610958 BD, HIF-2α – Rabbit –1∶500 Cat# ab20654 Abcam, β-Actin – Mouse 1∶10,000 Cat# ab8226 Abcam, GAPDH – Rabbit 1∶10,000 Cat# ab9485 Abcam, PDK1– Mouse 1∶1000 Cat# ab110025 Abcam, SOD2– Rabbit 1∶5000 Cat# ab13533 Abcam, VHL – Rabbit –1∶1000 Cat# ab28434 Abcam, and TNFR1– Rabbit –1∶1000 Cat# ab19139.

### Real Time q-PCR

RNA was extracted as previously described [22], reverse transcribed into cDNA performed using Superscript III first strand synthesis supermix (Invitrogen). cDNA was normalised against the reference gene 28s-RNA. QPCR was run in an ABI7500 real-time PCR system using Power SYBR green mix (Applied Biosystems). See supporting information file (Supporting Information S1) for conditions and full primer sequences.

### Flow Cytometry

Cells were analysed on a Beckton Dickenson (BD) LSR II and fluorescence-activated cell sorting (FACs) was performed on a BD Facs Aria. Cultured cells were detached using Accutase (PAA), re-suspended in PBS at 1×10^6^ cells/ml and incubated with the antibodies against CD44 (clone G44-26, BD biosciences) and ESA (clone HEA-125, Miltenyi Biotec) at 1∶100 for 15 mins. Cells were washed once before re-suspending in fresh PBS with DAPI (Sigma) at 200 ng/ml to exclude dead cells.

### Oxygen and ROS Measurements

The oxygen consumption of cell line fractions was measured in 96-well oxygen biosensor plates (BD Biosciences, Oxford, UK Cat# 353830) using the protocol adapted by Heywood et al [29], for quantitative measurements. ROS staining was carried out on sorted live cell fractions using CellROX™ (Invitrogen) at 5 mM together with Hoechst 33342 at 20 µg/ml for 30 mins as per manufacturer’s instructions. Quantification was obtained by flow cytometric analysis of cells triple stained for ESA, CD44 and CellROX™.

### Lactate and Glucose Assays

Lactate was determined as previously reported, with 1×10^5^ cells re-suspended in 100 µl of medium (phenol red free) and incubated for 8 hours. Lactate was calculated by comparison with a standard curve for lactate (Sigma L1750) ranging from 0–10 mM. Glucose concentrations were determined using a glucose colorimetric assay kit (Abcam, ab65333). Lactate fermentation fraction was calculated by comparison of expelled lactate with the theoretical maximum calculated for the glucose used.

### Mitochondrial Assays

Mitochondrial mass was assessed using MitoTracker Green™ (Invitrogen) according to manufacturer’s instructions. Cultures were grown from clonal density, detached from the dish. and incubated for 15 mins at 37°C with 50 nM MitoTracker (Green) before washing and analysing. For inner membrane mitochondrial potential (IMMP) cell suspensions were loaded with Dil_1_C (40 nM) (Invitrogen) and incubated at 37°C for 15 mins before washing once and re-suspending in fresh PBS. Mean values for the EMT and non-EMT CSC fractions were expressed as fractions of the values for the corresponding parental populations.

### Cell Cycle Analysis

For cell cycle analyses, cell suspensions were fixed in 70% ethanol, washed once in PBS and incubated in DAPI (1 ug/ml) (Sigma) for 20 mins together with antibodies against CD44 and ESA.

### Statistical Analysis

All experiments were repeated a minimum of 3 times and comparisons between values were performed using a two-tailed paired t-test unless otherwise stated. Error bars are reported as ±SEM.

## Results

### Long-term Hypoxia Promotes an EMT Phenotype and Increases a Subpopulation Characterised by Decreased ESA

**Figure 1:**
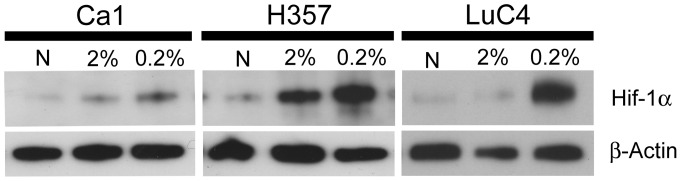
HNSCC cell lines express increased HIF-1α at lower oxygen concentrations. Western blot of protein lysates of Ca1, H357 and LuC4 cell lines after culture under normoxia (N), 2% and 0.2% oxygen.

**Figure 2:**
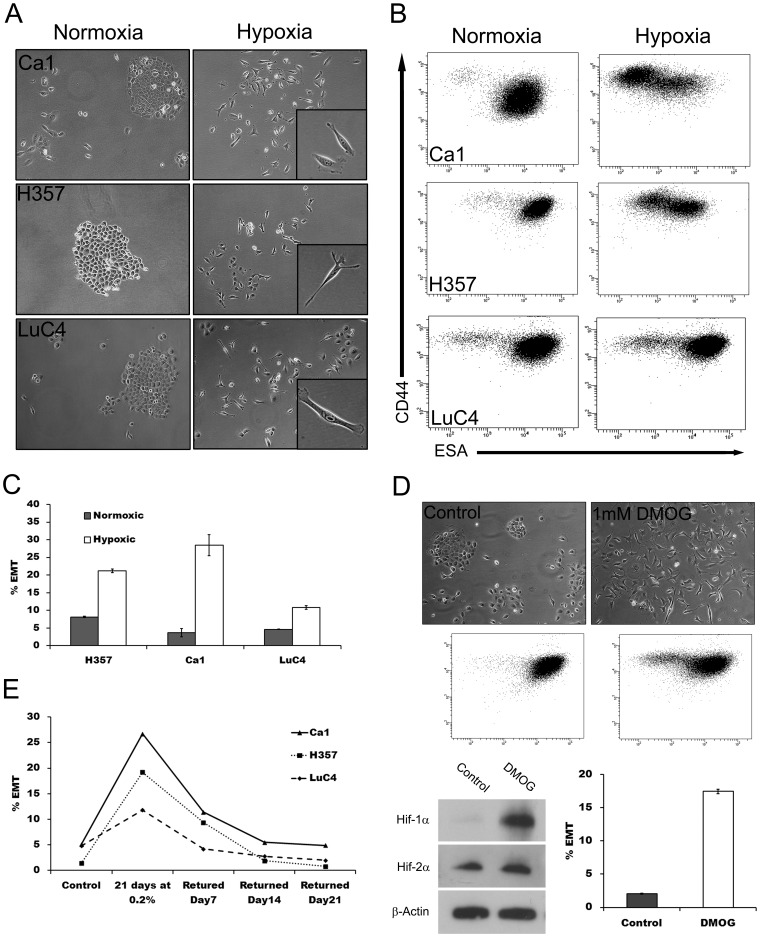
Hypoxia-induced changes in cell morphology and staining patterns consistent with EMT. **A**; phase-contrast images of Ca1, H357 and LuC4 cells grown under normoxic and 0.2% hypoxic culture conditions for 21 days (insets: higher magnification of EMT-like cells). **B**; cytometric analyses indicating a shift in staining for CD44 (y axis) and ESA (x axis) of cells resulting from hypoxia. **C**; analysis of the increases in EMT resulting from hypoxia. **D**; growth of the Ca1 cell line for 21 days with 1 mM DMOG results in morphological changes (top), corresponding changes in CD44 and ESA staining (middle), stabilisation HIF-1α but little change in HIF-2α (bottom left), and a increase in the EMT fraction (bottom right). **E**; time dependant decrease in the size of hypoxia-induced EMT fractions after return to normoxic conditions.

To assess cellular hypoxic responses to lowered oxygen concentrations, 3 HNSSC cell lines (Ca1, H357 and LuC4) were cultured in either normoxic (∼20% O_2_) or hypoxic (2% or 0.2%) levels of oxygen before preparing protein lysates to assess HIF-1α stabilization. All cell lines responded to hypoxia with increased HIF-1α protein (Fig. 1). To determine the effects of hypoxia on the size of EMT sub-populations [22], cells were cultured under hypoxia for up to 21 days and examined for morphological changes. Long-term cultures (14–21 days) exhibited a marked decrease in the number of cells forming cohesive colonies and an increase in the number of fibroblast-like cells (Fig. 2A). To establish whether this morphological change represented development of an EMT population, cells were analysed by flow cytometry for the CD44^high^/ESA^low^ EMT phenotype previously identified [22]. For all cell lines, long term hypoxia gave rise to a large increase in CD44^high^/ESA^low^ cell fractions (Fig. 2B, Fig. 2C). Increases in EMT were less at 2% than at 0.2% oxygen. The prolylhydroxylase inhibitor, DMOG, resulted in stabilisation of HIF-1α, and also some stabilization of HIF-2α (Fig. 2D). This was associated with an increase in cells with an EMT-like, appearance and the CD44^high^/ESA^low^ phenotype (Fig. 2D). To determine the reversibility of EMT upon removal from hypoxia, cells cultured under 0.2% oxygen for 21 days were returned to normoxic conditions and assessed by flow cytometry at 7 day intervals. In all lines, the size of the CD44^high^/ESA^low^ EMT fraction decreased over time and by 21 days had returned back to the levels of control cultures (Fig. 2E).

### Hypoxia Induces a Cell Population with EMT-related Gene Expression Patterns and Increased Sphere Forming Ability

**Figure 3:**
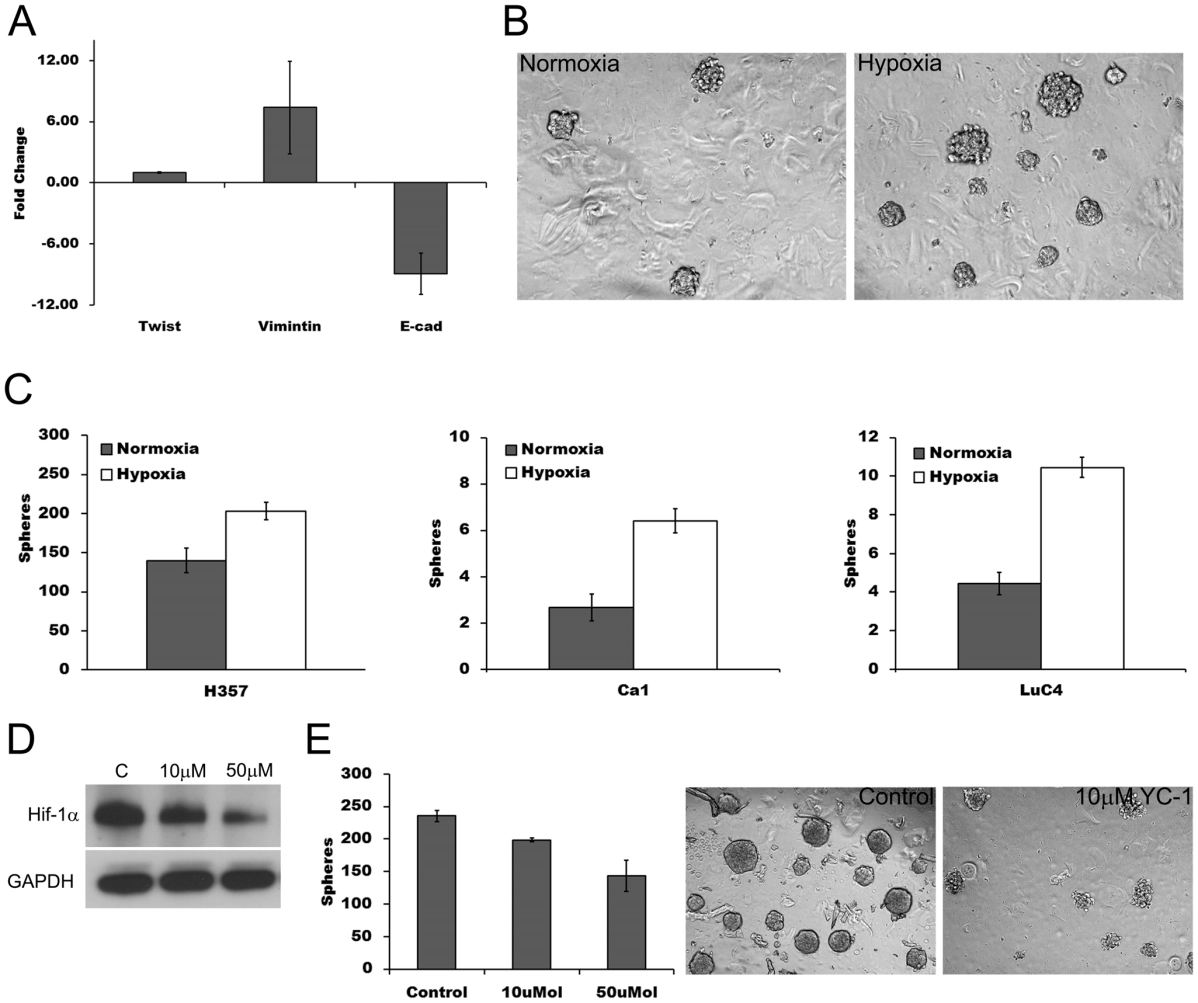
Long term hypoxic culture increases sphere forming ability. **A**; fold change in gene expression of EMT markers for CD44^high^/ESA^low^ cells relative to CD44^high^/ESA^high^ cells. **B**; representative samples of sphere forming cultures after 21 days of growth in normoxic and hypoxic conditions. **C**; increase in sphere formation following hypoxic culture. **D**; decrease in basal levels HIF-1α in normoxic cultures of H357 after addition of YC-1. **E**; decrease in normoxic sphere formation following addition of YC-1 (left) and phase microscopy (right) of control and YC-1 (10 µM) treated sphere cultures.

To determine whether the enlarged CD44^high^/ESA^low^ fraction induced by hypoxia had patterns of gene expression typical of EMT, subpopulations of Ca1 cells were FACS sorted before RNA extraction. Compared with the ESA^high^/CD44^high^ (Epi CSC) fraction, CD44^high^/ESA^low^ (EMT) fractions showed, as previously reported [22], greater expression of the EMT-related genes Twist and Vimentin and decreased expression of E-cadherin (Fig. 3A). Sphere forming assays, which have been used as surrogate assays for CSCs [30], were undertaken to determine whether the increase in EMT induced by hypoxia increased the number of cells with this stem cell trait. For all cell lines, unfractionated populations of cells maintained in hypoxic conditions for 21 days formed a greater number of spheres than cells grown under normoxic conditions (Fig. 3B & 3C). The HIF-1α inhibitor YC-1 reduced basal levels of HIF-1α under normoxic conditions (Fig. 3D) and decreased of sphere forming ability (Fig. 3E).

### EMT Sub-fractions have Increased HIF-1α and Up-regulation of Anaerobic Metabolic Genes

**Figure 4:**
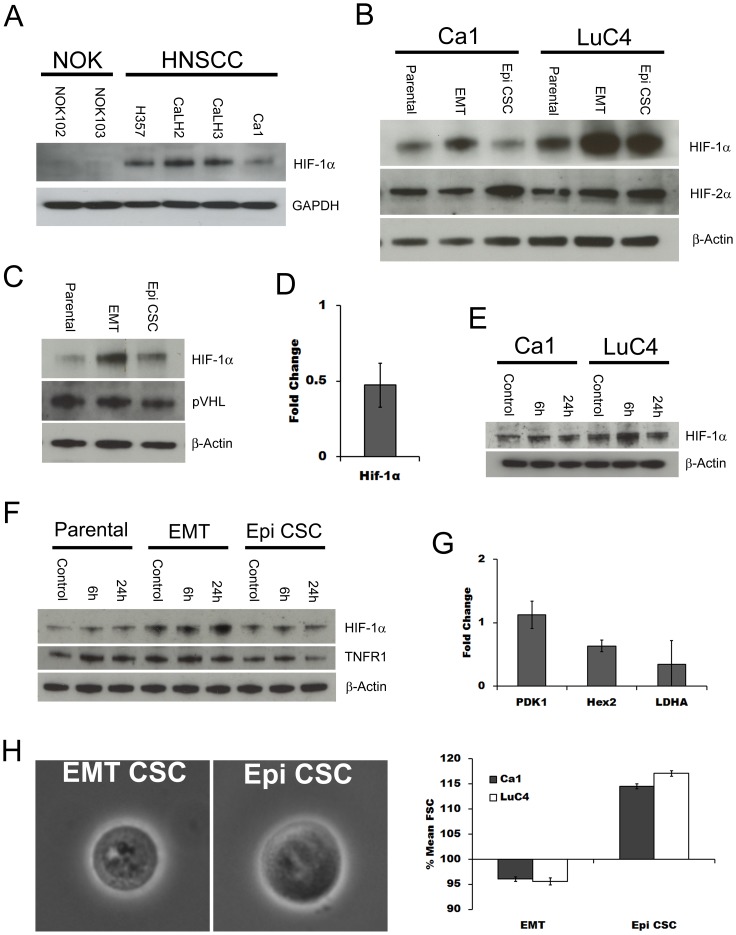
EMT cells have increased expression of HIF-1α. **A**; comparison by western blotting of normoxic expression of HIF-1α in NOK and HNSCC cell lines. **B**; HIF-1α and HIF-2α expression in sorted parental, EMT CSC and Epi CSC fractions of the Ca1 and LuC4 cell lines under normoxia. **C**; western blot for pVHL levels in sorted fractions of the Ca1 cell line. **D;** PCR evaluation of HIF-1α expressed as EMT CSC levels relative to Epi CSC levels for the Ca1 cell line. **E;** response of HIF-1α levels of Ca1 and LuC4 lines to treatment with 10 ng/ml TNFα for 6 or 24 hours under normoxic conditions. **F;** western blots for for HIF-1α and TNFR1 in sub fractions of the Ca1 cell line following TNFα treatment. **G;** PCR evaluation of glycolytic gene targets expressed as EMT CSC levels relative to Epi CSC levels within the Ca1 cell line. **H;** representative phase microscopic images of Epi and EMT cells (left) and comparison of forward scatter for EMT and Epi CSC fractions (right).

Under normoxic conditions, cancer cell lines, like their tumours of origin, usually show an increased proportion of energy derived from glycolytic processes together with higher levels of HIF-1α [5]. Normoxic cultures of HNSCC cell lines showed higher basal levels of HIF-1α than NOK (Fig. 4A). The further increase in EMT cells induced by hypoxia suggested that HIF-1α expression might differ between CSC sub-fractions. EMT and Epi CSC fractions were therefore sorted from the Ca1 and LuC4 cell lines for comparison with their unfractionated populations. Compared with Epi CSCs or the parental populations, EMT cells showed higher levels of HIF-1α whereas HIF-2α was higher in the Epi CSC fraction (Fig. 4B). As the higher levels of HIF-1α in EMT fractions could be produced by either reduced degradation or increased production of the protein,we assessed the Von Hippel-Lindau protein (pVHL) that targets HIF-1α for proteasomal degradation. However, no difference was found between fractions for pVHL protein levels (Fig 4C). Examining HIF-1α production within the EMT fraction we found that message for HIF-1α was elevated (Fig. 4D) suggesting that increased production rather than decreased degradation leads to the elevated levels seen in EMT cells. Production of HIF-1α under normoxic conditions is increased in response to the inflammatory cytokine TNFα, [31] and this cytokine is also a potent inducer of EMT [32]. To assess the effects of TNFα on the HIF-1α within our lines cell lines under normoxic conditions, cells were cultured with TNFα for 6 or 24 hours. At both time points, and in both lines tested, elevated levels of HIF-1α were seen (Fig. 4E). We then investigated whether the Epi and EMT sub-fractions responded differentially to TNFα and found that the higher baseline levels of HIF-1α present in EMT fractions were raised even further by TNFα (Fig 4F). No effect on the Epi CSC subpopulation was seen indicating a selectively effect on the EMT fraction, perhaps due to the higher amounts of the TNFα receptor 1 (TNFR1) expressed by these cells.

To assess whether the higher HIF-1α levels present within EMT cells under normoxic conditions correlated with increased expression of HIF-1 downstream genes, 3 glycolytic targets were examined. Hexokinase II (Hex2), pyruvate dehydrogenase kinase 1 (PDK1), and Lactate dehydrogenase A (LDHA), were all found to be increased in the EMT fraction compared to the Epi CSC fraction (Fig. 4G). As PDK1 is reported to influence the proportion of glucose used for the fermentation to lactate we investigated whether the observed increases in HIF-1α and its target glycolytic genes correlate with changes in glucose metabolism and the production of lactate. No differences were seen in PDK1 protein levels between fractions (Fig. S1A) and examination of glucose consumption and lactate production revealed a similar proportion of glucose anaerobically metabolised for each fraction, regardless of HIF-1α expression (Fig. S1B & C). However total energy demand may be related to cell size and EMT CSCs were found to be smaller than Epi CSCs, an observation confirmed by forward scatter analysis (Fig. 4H).

### Oxygen Consumption is Greatly Reduced in Normoxic EMT Cells and is Associated with Altered Cell Cycle, Reduction in ROS, and Mitochondrial Changes

**Figure 5:**
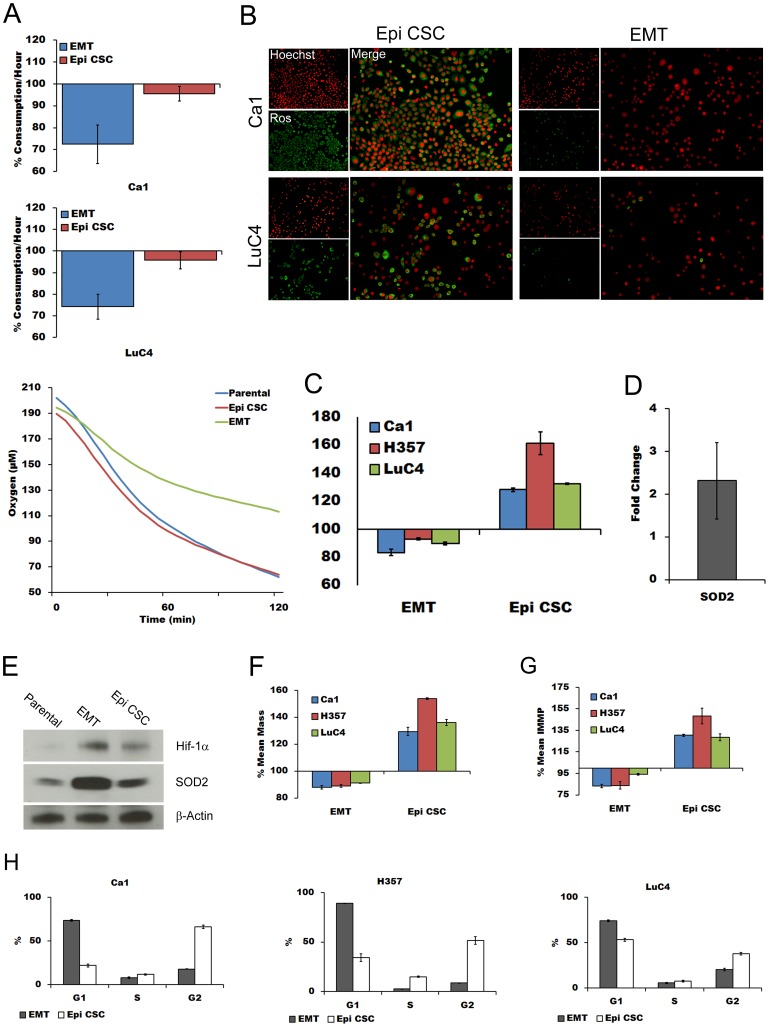
EMT CSCs have reduced oxygen consumption, less ROS, reduced mitochondrial mass and inner membrane potential, increased SOD2 and an altered cell cycle. **A**; relative oxygen consumption of EMT and Epi CSC fractions compared to that of parental line for the first hour (top panels) and for Ca1 over 2 hours (below). **B**; immunofluorescent images comparing ROS levels in EMT and Epi CSCs counterstained with Hoechst. **C**; flow cytometric analyses of mean ROS levels of EMT and Epi CSCs relative to parental cells. **D**; PCR fold differences for SOD2 between EMT and Epi CSCs. **E**; western blots for HIF-1α and SOD2 for the 3 sub-fractions of Ca1 cell line. **F**; mean mitochondrial mass of EMT and Epi CSCs relative to parental cells. **G**; comparison of IMMP of EMT and Epi CSCs relative to parental cells. **H**; cell cycle profiles for EMT and Epi CSC fractions of the 3 cell lines.

Hypoxic up-regulation of PDK1 by HIF1 affects mitochondrial function, oxygen metabolism and ROS production [6], [33]. To assess whether the increased levels of HIF-1α present in normoxic EMT fractions correlate with the HIF-1α functions in hypoxic fractions, sorted sub-fractions of normoxic cultures were assayed for oxygen consumption. During the first hour, and persisting for the duration of the assay (Fig. 5A), the consumption of oxygen by the EMT fractions of both the Ca1 and LuC4 cell lines was approximately 25% less than either the Epi CSC fractions or parental populations.

To establish if reduced oxygen consumption correlated with the reduced ROS levels of EMT cells, live EMT and Epi CSC cells were compared using the ROS marker CellRox™ Deep Red Reagent. Much less staining for this marker was seen for EMT cells (Fig. 5B) and cytometric quantification of ROS in cells triple stained for ESA, CD44 and CellRox™ confirmed less ROS in the EMT CSC population (Fig. 5C). QPCR (Fig. 5D). Western blotting (Fig 5E) showed the ROS scavenger superoxide dismutase 2 (SOD2) to be highly expressed within EMT CSCs.

As oxidative phosphorylation occurs within mitochondria, it was anticipated that altered oxygen consumption might be related to altered mitochondrial states within CSC sub-populations. Differences in total mitochondrial mass between cell sub-populations were therefore assessed by triple-staining for ESA, CD44 and MitoTracker Green™. For all cell lines, EMT fractions showed significantly less mitochondrial mass (∼10%) than the parental population and a striking 40% reduction compared to the Epi CSC population (Fig. 5F). As a marker of functon, inner mitochondrial membrane potential (IMMP) was assessed by loading cells with Dil_1_C(5). This showed a lower IMMP (∼10%) for EMT fractions than parental populations and an even lower level compared with Epi CSCs (Fig. 5G). Assessment of cell cycle differences between cell fractions indicated that Epi CSCs tended to accumulate in G2, as previously shown [18], whereas the majority of EMT cells were present in G1 (Fig. 5H).

## Discussion

We have recently described heterogeneity within the CSC sub-population [22] and that while the majority of CSCs have a CD44^high^/ESA^high^ cell surface phenotype and express epithelial markers, a variably sized fraction of CSCs has undergone EMT. Both the Epi and EMT CSC populations are self-renewing *in vitro* and are tumour initiating if transplanted *in vivo* (25). However, EMT CSCs are resistant to anoikis and have greater ability to grow in suspension as tumour spheres, a property that provides a surrogate assay for this population. Assessed either morphologically or as CD44^high^/ESA^low^ cells, the small EMT fraction typically present in cell lines under normoxic conditions increases greatly in response to hypoxia or after stabilization of HIF-1α protein, changes consistent with previous reports [23]–[26]. This enlarged population displays a gene expression profile typical of EMT and has enhanced sphere-forming ability. After accelerated degradation of HIF-1α there is a reduction in sphere formation. Taken together, these findings indicate that HIF-1α has a functional role in inducing and maintaining the CSC EMT phenotype.

HIF-1α is recognised to drive cells towards a metabolic phenotype with reduced oxidative metabolism and increased lactate production, mediated in part by the up-regulation of PDK-1 [6], [33]. Under normoxic conditions, we find an approximately 50% greater rate of synthesis of HIF-1α within the EMT fraction and that this correlated with higher protein levels. Similar levels of pVHL protein suggested no change in the rate of degradation and we therefore conclude that the higher level of HIF-1α within EMT is maintained by increased production. EMT is reported to be induced by TGF-β1, interleukins and inflammatory cytokines such as TNFα, which has also been reported to increase production of HIF-1α [31]. We have confirmed similar responses of our cell lines and also show that EMT cells respond selectively to TNFα by increasing their level of HIF-1α. This importantly implicates effects of the tumour microenviroment in shifting and maintaining the EMT phenotype and therefore the progression and invasion of tumours.

HIF-1α production has also been suggested due to a feed-forward loop with PDK-1 [34]. However although we found increased message for PDK-1 within the EMT CSC sub-population we found no change in protein levels. Also no difference was found between the epithelial and EMT fractions in the proportion of glucose metabolised to lactate which was about 60% for all subpopulations, a percentage similar to that reported by Warburg over 80 years ago [1]. However, proportional to EMT CSCs size, the amount of glucose usage would be higher and this taken together with the marked reduction in oxygen consumption, suggests a reduced dependence on oxidative phosphorylation with EMT cells being less metabolically active than Epi CSCs. The cell cycle changes found are also suggestive of a more quiescent EMT phenotype as has previously shown for EMT cells in HNSCC lines [35].

Under hypoxia, HIF1 acting through PDK1 [6] leads to a reduction in mitochondrial oxygen consumption [33] and reduced production of ROS. Over-expression of PDK1 also correlates with poor prognosis [36]. Higher expression levels of HIF-1α and PDK1 were found within the normoxic CSC EMT population accompanied by a reduction in oxygen consumption and lower ROS levels, changes similar to those reported for cells under hypoxic conditions. The high levels of SOD2 in EMT CSCs would also function to inactivate such ROS as might be produced. Papandreou and co-workers [33], examining unfractionated tumour populations, detected no structural changes in mitochondria. However, analysing differences between CSC sub-fractions showed a reduction in mitochondrial mass in EMT CSCs together with a lower relative IMMP which points to reduced mitochondrial function. Zhang et al [37] have reported repressive actions of HIF1 on mitochondrial biogenesis through repression of transcription by C-Myc [38], [39] and we have previously reported down-regulation of C-Myc in the normoxic EMT sub-population [22]. PCR analyses (data not shown) also indicate down regulation of C-Myc within the enlarged hypoxic EMT fractions.

CSCs have several unique properties that distinguish them from the bulk of the differentiating cell population [12], [18], [30] and recent publications have associated acquisition of stem cell properties with EMT. For example, induction of EMT in a non-tumorigenic breast cell line by treatment with TGF-β1 leads to acquisition of the CD44^high^/CD24^low^ cell surface phenotype typical of breast CSCs that have tumour-initiating and tumour-sphere forming abilities [16]. Radiation therapy is widely used for treatment of HNSCC and low levels of oxygen and cellular ROS, together with higher levels of antioxidants, are critical mediators of reduced cell killing by irradiation [12]. Low oxygen levels are associated with radiotherapy failure [40] and it is therefore of particular clinical interest that hypoxic induction of CSCs into the EMT phenotype would increase yet further the general resistance to various therapeutic modalities shown by CSCs [17]. The present data extends these findings, first by supporting the presence, as previously reported [22], of two stem cell phenotypes and, secondly, by showing several additional properties of the EMT CSC phenotype that are likely to be associated with enhanced therapeutic resistance. The metabolic properties of the EMT CSC fraction, and its increase in size in hypoxia, are likely therefore to act to enhance radiation resistance. Further, the slower cell cycle and mitochondrial reduction in EMT CSCs may also have implications for apoptotic responses to both radiation and anti-cancer drugs. It appears that therapeutic elimination of the less metabolically active EMT population will require targeted treatment and that the models of EMT induction, through both hypoxia and cytokines may provide a crucial platform for development of such therapies.

## Supporting Information

Figure S1 **Sub fraction glucose utilisation. A;** western blots for PDK1 levels of sub fractions. **B;** glucose usage (left), lactate expelled (center) and percent of glucose metabolised to form lactate (right) in the Ca1 cell line and **C;** in the LuC4 cell line.
(TIF)

Supporting Information S1 **QPCR Conditions and Primers.**
(DOC)

## References

[1] Warburg O, Wind F, Negelein E (1927) The Metabolism of Tumors in the Body. J Gen Physiol 8: 519–530. 19872213 10.1085/jgp.8.6.519 PMC2140820

[2] Warburg O (1956) On respiratory impairment in cancer cells. Science 124: 269–270. 13351639

[3] Kallio PJ, Pongratz I, Gradin K, McGuire J, Poellinger L (1997) Activation of hypoxia-inducible factor 1alpha: posttranscriptional regulation and conformational change by recruitment of the Arnt transcription factor. Proc Natl Acad Sci U S A 94: 5667–5672. 9159130 10.1073/pnas.94.11.5667 PMC20836

[4] Semenza GL (2004) Hydroxylation of HIF-1: oxygen sensing at the molecular level. Physiology (Bethesda) 19: 176–182. 15304631 10.1152/physiol.00001.2004

[5] Zhong H, De Marzo AM, Laughner E, Lim M, Hilton DA, et al (1999) Overexpression of hypoxia-inducible factor 1alpha in common human cancers and their metastases. Cancer Res 59: 5830–5835. 10582706

[6] Kim JW, Tchernyshyov I, Semenza GL, Dang CV (2006) HIF-1-mediated expression of pyruvate dehydrogenase kinase: a metabolic switch required for cellular adaptation to hypoxia. Cell Metab 3: 177–185. 16517405 10.1016/j.cmet.2006.02.002

[7] Ayrapetov MK, Xu C, Sun Y, Zhu K, Parmar K, et al (2011) Activation of Hif1alpha by the prolylhydroxylase inhibitor dimethyoxalyglycine decreases radiosensitivity. PLoS One 6: e26064. 22016813 10.1371/journal.pone.0026064 PMC3189247

[8] Sasabe E, Zhou X, Li D, Oku N, Yamamoto T, et al (2007) The involvement of hypoxia-inducible factor-1alpha in the susceptibility to gamma-rays and chemotherapeutic drugs of oral squamous cell carcinoma cells. Int J Cancer 120: 268–277. 17066447 10.1002/ijc.22294

[9] Wartenberg M, Ling FC, Muschen M, Klein F, Acker H, et al (2003) Regulation of the multidrug resistance transporter P-glycoprotein in multicellular tumor spheroids by hypoxia-inducible factor (HIF-1) and reactive oxygen species. FASEB J 17: 503–505. 12514119 10.1096/fj.02-0358fje

[10] Sasabe E, Tatemoto Y, Li D, Yamamoto T, Osaki T (2005) Mechanism of HIF-1alpha-dependent suppression of hypoxia-induced apoptosis in squamous cell carcinoma cells. Cancer Sci 96: 394–402. 16053510 10.1111/j.1349-7006.2005.00065.x PMC11158431

[11] Sullivan R, Pare GC, Frederiksen LJ, Semenza GL, Graham CH (2008) Hypoxia-induced resistance to anticancer drugs is associated with decreased senescence and requires hypoxia-inducible factor-1 activity. Mol Cancer Ther 7: 1961–1973. 18645006 10.1158/1535-7163.MCT-08-0198

[12] Diehn M, Cho RW, Lobo NA, Kalisky T, Dorie MJ, et al (2009) Association of reactive oxygen species levels and radioresistance in cancer stem cells. Nature 458: 780–783. 19194462 10.1038/nature07733 PMC2778612

[13] Clarke MF, Dick JE, Dirks PB, Eaves CJ, Jamieson CH, et al (2006) Cancer stem cells–perspectives on current status and future directions: AACR Workshop on cancer stem cells. Cancer Res 66: 9339–9344. 16990346 10.1158/0008-5472.CAN-06-3126

[14] Brabletz T, Jung A, Spaderna S, Hlubek F, Kirchner T (2005) Opinion: migrating cancer stem cells - an integrated concept of malignant tumour progression. Nat Rev Cancer 5: 744–749. 16148886 10.1038/nrc1694

[15] Thiery JP (2003) Epithelial-mesenchymal transitions in development and pathologies. Curr Opin Cell Biol 15: 740–746. 14644200 10.1016/j.ceb.2003.10.006

[16] Mani SA, Guo W, Liao MJ, Eaton EN, Ayyanan A, et al (2008) The epithelial-mesenchymal transition generates cells with properties of stem cells. Cell 133: 704–715. 18485877 10.1016/j.cell.2008.03.027 PMC2728032

[17] Gupta PB, Onder TT, Jiang G, Tao K, Kuperwasser C, et al (2009) Identification of selective inhibitors of cancer stem cells by high-throughput screening. Cell 138: 645–659. 19682730 10.1016/j.cell.2009.06.034 PMC4892125

[18] Harper LJ, Costea DE, Gammon L, Fazil B, Biddle A, et al (2010) Normal and malignant epithelial cells with stem-like properties have an extended G2 cell cycle phase that is associated with apoptotic resistance. BMC Cancer 10: 166. 20426848 10.1186/1471-2407-10-166 PMC2868812

[19] Harper LJ, Piper K, Common J, Fortune F, Mackenzie IC (2007) Stem cell patterns in cell lines derived from head and neck squamous cell carcinoma. J Oral Pathol Med 36: 594–603. 17944752 10.1111/j.1600-0714.2007.00617.x

[20] Al-Hajj M, Wicha MS, Benito-Hernandez A, Morrison SJ, Clarke MF (2003) Prospective identification of tumorigenic breast cancer cells. Proc Natl Acad Sci U S A 100: 3983–3988. 12629218 10.1073/pnas.0530291100 PMC153034

[21] Prince ME, Sivanandan R, Kaczorowski A, Wolf GT, Kaplan MJ, et al (2007) Identification of a subpopulation of cells with cancer stem cell properties in head and neck squamous cell carcinoma. Proc Natl Acad Sci U S A 104: 973–978. 17210912 10.1073/pnas.0610117104 PMC1783424

[22] Biddle A, Liang X, Gammon L, Fazil B, Harper LJ, et al.. (2011) Cancer stem cells in squamous cell carcinoma switch between two distinct phenotypes that are preferentially migratory or proliferative. Cancer Res. 10.1158/0008-5472.CAN-11-1059 21685475

[23] Yang MH, Wu MZ, Chiou SH, Chen PM, Chang SY, et al (2008) Direct regulation of TWIST by HIF-1alpha promotes metastasis. Nat Cell Biol 10: 295–305. 18297062 10.1038/ncb1691

[24] Yang MH, Hsu DS, Wang HW, Wang HJ, Lan HY, et al (2010) Bmi1 is essential in Twist1-induced epithelial-mesenchymal transition. Nat Cell Biol 12: 982–992. 20818389 10.1038/ncb2099

[25] Louie E, Nik S, Chen JS, Schmidt M, Song B, et al (2010) Identification of a stem-like cell population by exposing metastatic breast cancer cell lines to repetitive cycles of hypoxia and reoxygenation. Breast Cancer Res 12: R94. 21067584 10.1186/bcr2773 PMC3046435

[26] Lester RD, Jo M, Montel V, Takimoto S, Gonias SL (2007) uPAR induces epithelial-mesenchymal transition in hypoxic breast cancer cells. J Cell Biol 178: 425–436. 17664334 10.1083/jcb.200701092 PMC2064849

[27] Prime SS, Nixon SV, Crane IJ, Stone A, Matthews JB, et al (1990) The behaviour of human oral squamous cell carcinoma in cell culture. J Pathol 160: 259–269. 1692339 10.1002/path.1711600313

[28] Gemenetzidis E, Bose A, Riaz AM, Chaplin T, Young BD, et al (2009) FOXM1 upregulation is an early event in human squamous cell carcinoma and it is enhanced by nicotine during malignant transformation. PLoS One 4: e4849. 19287496 10.1371/journal.pone.0004849 PMC2654098

[29] Heywood HK, Bader DL, Lee DA (2006) Rate of oxygen consumption by isolated articular chondrocytes is sensitive to medium glucose concentration. J Cell Physiol 206: 402–410. 16155906 10.1002/jcp.20491

[30] Dontu G, Abdallah WM, Foley JM, Jackson KW, Clarke MF, et al (2003) In vitro propagation and transcriptional profiling of human mammary stem/progenitor cells. Genes Dev 17: 1253–1270. 12756227 10.1101/gad.1061803 PMC196056

[31] van Uden P, Kenneth NS, Rocha S (2008) Regulation of hypoxia-inducible factor-1alpha by NF-kappaB. Biochem J 412: 477–484. 18393939 10.1042/BJ20080476 PMC2474706

[32] Wang H, Wang HS, Zhou BH, Li CL, Zhang F, et al (2013) Epithelial-Mesenchymal Transition (EMT) Induced by TNF-alpha Requires AKT/GSK-3beta-Mediated Stabilization of Snail in Colorectal Cancer. PLoS One 8: e56664. 23431386 10.1371/journal.pone.0056664 PMC3576347

[33] Papandreou I, Cairns RA, Fontana L, Lim AL, Denko NC (2006) HIF-1 mediates adaptation to hypoxia by actively downregulating mitochondrial oxygen consumption. Cell Metab 3: 187–197. 16517406 10.1016/j.cmet.2006.01.012

[34] McFate T, Mohyeldin A, Lu H, Thakar J, Henriques J, et al (2008) Pyruvate dehydrogenase complex activity controls metabolic and malignant phenotype in cancer cells. J Biol Chem 283: 22700–22708. 18541534 10.1074/jbc.M801765200 PMC2504897

[35] Chen C, Wei Y, Hummel M, Hoffmann TK, Gross M, et al. Evidence for epithelial-mesenchymal transition in cancer stem cells of head and neck squamous cell carcinoma. PLoS One 6: e16466. 10.1371/journal.pone.0016466 PMC3029362 21304586

[36] Wigfield SM, Winter SC, Giatromanolaki A, Taylor J, Koukourakis ML, et al (2008) PDK-1 regulates lactate production in hypoxia and is associated with poor prognosis in head and neck squamous cancer. Br J Cancer 98: 1975–1984. 18542064 10.1038/sj.bjc.6604356 PMC2441961

[37] Zhang H, Gao P, Fukuda R, Kumar G, Krishnamachary B, et al (2007) HIF-1 inhibits mitochondrial biogenesis and cellular respiration in VHL-deficient renal cell carcinoma by repression of C-MYC activity. Cancer Cell 11: 407–420. 17482131 10.1016/j.ccr.2007.04.001

[38] Sutphin PD, Giaccia AJ, Chan DA (2007) Energy regulation: HIF MXIes it up with the C-MYC powerhouse. Dev Cell 12: 845–846. 17543856 10.1016/j.devcel.2007.05.006

[39] Li Q, Kluz T, Sun H, Costa M (2009) Mechanisms of c-myc degradation by nickel compounds and hypoxia. PLoS One 4: e8531. 20046830 10.1371/journal.pone.0008531 PMC2797325

[40] Brizel DM, Dodge RK, Clough RW, Dewhirst MW (1999) Oxygenation of head and neck cancer: changes during radiotherapy and impact on treatment outcome. Radiother Oncol 53: 113–117. 10665787 10.1016/s0167-8140(99)00102-4

