# Supplementary figures and images for: Sub-Sets of Cancer Stem Cells Differ Intrinsically in Their Patterns of Oxygen Metabolism
Source: PLoS One. 2013 Apr 30;8(4):e62493. doi: 10.1371/journal.pone.0062493 (PMC3640080; doi:10.1371/journal.pone.0062493)

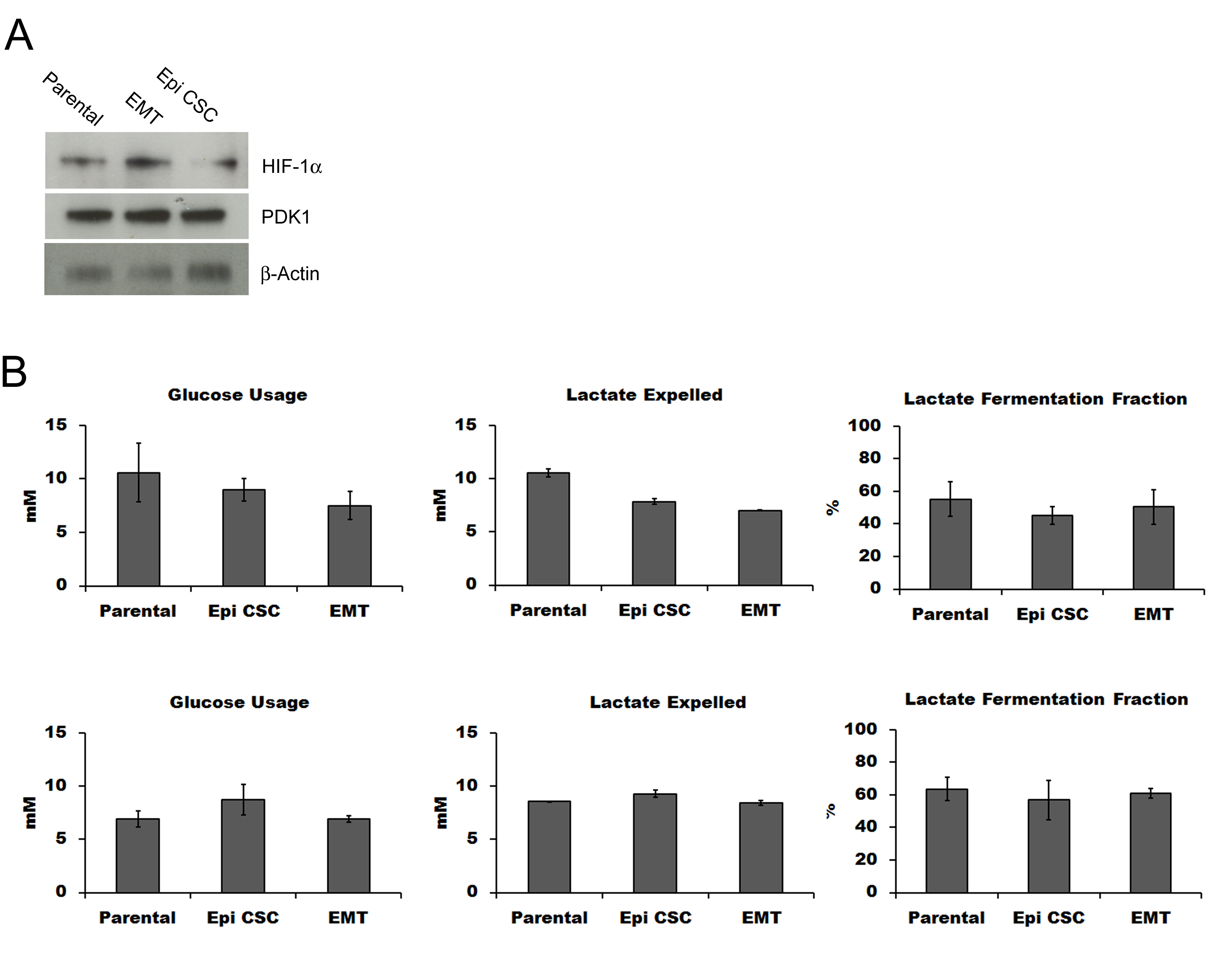

Supplement: Figure S1 — Sub fraction glucose utilisation. A; western blots for PDK1 levels of sub fractions. B; glucose usage (left), lactate expelled (center) and percent of glucose metabolised to form lactate (right) in the Ca1 cell line and C; in the LuC4 cell line. (TIF) [file pone.0062493.s001.tif]
