# Supplementary material for: Sub-Sets of Cancer Stem Cells Differ Intrinsically in Their Patterns of Oxygen Metabolism
Source: PLoS One. 2013 Apr 30;8(4):e62493. doi: 10.1371/journal.pone.0062493 (PMC3640080; doi:10.1371/journal.pone.0062493)
Supplement: Supporting Information S1 — QPCR Conditions and Primers. (DOC) [file pone.0062493.s002.doc]

**Supplementary Info**

**QPCR conditions**

QPCR cycling conditions were: 95°C for 10mins, [95°C for 15 seconds, 60°C for 30 seconds, 72°C for 40 seconds] (40 cycles), 95°C for 60 seconds, followed by dissociation curve analysis. Reverse transcribed Human Total Reference RNA (Stratagene) was used to generate a standard curve.

**Primers for QPCR**

| **Gene** | **Abbrv** | **Forward** | **Reverse** |
| --- | --- | --- | --- |
| **28S ribosomal RNA** | **28S rRNA** | **GCCGGGGGCCTCCCACTTAT** | **TGGCGGAATCAGCGGGGAAA** |
| **C-myc** | **C-myc** | **GCTGCCAGGACCCGCTTCTC** | **ACGTTGAGGGGCATCGTCGC** |
| **E-cadherin** | **E-cad** | **GAACGCATTGCCACATACAC** | **AGCACCTTCCATGACAGACC** |
| **Hexokinase 2** | **HEX II** | **TCGTTCCCCTGCCACCAGACT** | **TGGCCTTCCGGATCAGAGCCA** |
| **Hif1α** | **Hif1α** | **CCGCTGGAGACACAATCATA** | **GGTGAGGGGAGCATTACATC** |
| **Lactate dehydrogenase A** | **LDHA** | **TGGGGTTGGTGCTGTTGGCA** | **GGAAAAGGCTGCCATGTTGGAGA** |
| **Pyruvate Dehydrogenase Kinase 1** | **PDK1** | **TGGTGGAAAAGGCAAAGGAAGTC** | **CAGACGCCTAGCATTTTCATAGC** |
| **Superoxide dismutase 2** | **SOD2** | **AGGCTCAGGTTGGGGTTGGCT** | **GCGTGCTCCCACACATCAATCCC** |
| **Twist** | **Twist** | **GTCCGCAGTCTTACGAGGAG** | **CCAGCTTGAGGGTCTGAATC** |
| **Vimentin** | **Vim** | **CCCTCACCTGTGAAGTGGAT** | **GACGAGCCATTTCCTCCTTC** |
